# 6–18 GHz High-Efficiency Power Amplifier MMIC Based on Broadband Impedance Matching

**DOI:** 10.3390/mi17060690

**Published:** 2026-06-03

**Authors:** Shuai Liu, Xiaohua Ma, Yi Zhang, Zhaoke Bian, Chunliang Xu

**Affiliations:** 1 School of Microelectronics, Xidian University, Xi’an 710071, China; xhma@xidian.edu.cn; 2 Microwave Application Research Institute, The 13th Research Institute of China Electronics Technology Group Corporation, Shijiazhuang 050051, China; zhangyi1026@foxmail.com (Y.Z.); 18729181180@163.com (Z.B.); mrxcl@163.com (C.X.)

**Keywords:** power amplifier, broadband power amplifier, high efficiency, Chebyshev matching network

## Abstract

To meet the high standard requirements for broadband high-efficiency power amplifiers in modern communication technology, a 6–18 GHz high-efficiency monolithic microwave integrated circuit (MMIC) power amplifier was developed using a 0.25 μm gallium nitride high-electron mobility transistor (GaN HEMT) process. A multistage Chebyshev-filter-based matching approach is utilized to provide the requisite bandwidth while concurrently managing second-harmonic terminations for enhanced PAE. In the final power stage, a multi-cell combining architecture is employed to achieve high saturated output power. The designed GaN amplifier achieves a saturated power of above 43.5 dBm and a PAE of over 30%. The area of the proposed GaN amplifier is 4 × 3.2 mm^2^. This chip, with its high efficiency and compact size, is promising for high-performance wideband systems.

## 1. Introduction

High-performance microwave systems—ranging from next-generation 5G infrastructure to advanced radar and satellite links—are driving a shift toward more capable microwave circuit technologies. As system bandwidths expand and power requirements continue to rise, the RF power amplifier (PA) has become a critical bottleneck. Modern RF front ends demand PAs that simultaneously provide wide bandwidth, high efficiency, and substantial output power. The rapid proliferation of modern communication standards continues to drive demand for RF power amplifiers (PAs) capable of operating across multiple frequency bands and modulation formats. To meet the requirements of these multi-octave applications, several circuit topologies have been explored, including low-frequency push–pull stages [1,2], feedback amplifiers [3,4,5], distributed amplifiers (DAs) [6,7] capable of multi-octave operation, balanced architectures, and reactively matched power amplifiers (RMPAs). Many high-performance, broadband GaN multi-octave power amplifiers (HPAs) covering the 6–18 GHz band have been reported in the literature.

Recent studies have demonstrated the feasibility of ultra-wideband GaN-based MMIC power amplifiers (PAs) for multifunction radar, electronic warfare, and broadband communication systems. In early work, researchers primarily adopted multi-stage architectures to achieve broadband gain and output power. For instance, three-stage GaN MMIC HPAs operating over 6–18 GHz and fabricated in 0.25 µm GaN-on-SiC processes delivered 6–10 W of output power with an over 18 dB gain across the band and about 10 W of output power with ~20% PAE at 18 GHz [8], establishing the foundation for wideband GaN MMIC PA designs. To enhance power capacity and broadband robustness, balanced and push–pull architectures were subsequently explored. An integrated GaN MMIC chipset reported in ref. [9] combined a driver amplifier and a balanced high-power amplifier, achieving over 10 W of chip-level output power and up to 18 W at the module level across 6–18 GHz. In addition, a low-loss balun-based push–pull GaN MMIC PA demonstrated an output power above 41 dBm with 20–26% efficiency and improved even-order harmonic suppression over the same band [10]. Compact multi-way power-combining techniques have been investigated to further boost output power without sacrificing bandwidth. A four-way combined GaN MMIC PA employing shared drain- and gate-line architectures achieved over 20 W of average CW output power with approximately 10 dB gain across 6–18 GHz, demonstrating a state-of-the-art power density among octave-band GaN MMIC PAs extending though the Ku band [11]. Distributed power amplifier (DPA) architectures have also been extended toward multi-octave operation by incorporating reactively matched gain cells. As reported in ref. [12], GaN DPAs implemented in a 0.25 µm process achieved an output power exceeding 40 dBm, a peak PAE approaching 30%, and a gain above 15 dB over multi-octave bandwidths while maintaining a relatively compact die area. In parallel, advanced broadband-matching and harmonic management techniques have been introduced to further enhance efficiency and spectral performance. Elliptic-filter-based output-matching networks enabled over 41 dBm of output power with up to 40% PAE and strong harmonic suppression across 6–18 GHz [13], while non-Foster matching techniques demonstrated watt-level output power over multi-octave bandwidths by compensating for device parasitics [14]. Moreover, high-order Chebyshev-filter-based matching and power synthesis methods have been shown to realize simultaneous broadband impedance transformation and multi-way power combination, achieving over 16 W of output power with an over 25 dB gain across a 5.5–18 GHz bandwidth [15].

Motivated by the abovementioned studies, this paper presents a broadband high-power GaN MMIC power amplifier. A broadband impedance-matching technique is employed to enable low-loss and broadband impedance transformation. To maximize the power-added efficiency (PAE) over a broad frequency range, the Chebyshev matching network at the output stage was co-optimized with second-harmonic impedance engineering to realize low-loss broadband impedance transformation. By ensuring that the second-harmonic impedance remains within an efficiency-enhancing region over the entire 6–18 GHz band, the proposed approach simultaneously achieves wideband operation and high power-added efficiency. This ensures highly efficient operation and consistent power combination across the 6–18 GHz frequency spectrum. Fabricated in a 0.25 μm GaN HEMT process, the proposed power amplifier (PA) operates over the 6–18 GHz band, achieving a small-signal gain of up to 28 dB and a saturated output power of 44 dBm. Notably, the measured power-added efficiency (PAE) exceeds 30% across the entire operating band, with a peak value of 41%, while maintaining a compact chip area and robust broadband stability. To the best of our knowledge, this work demonstrates one of the highest efficiency levels reported for 6–18 GHz GaN power amplifiers while simultaneously maintaining a high output power. This simultaneous achievement effectively mitigates the conventional trade-off between efficiency and output power—with prior designs typically optimizing one metric at the expense of the other—thereby resulting in a clear performance enhancement over previously reported works. Consequently, these results indicate that the proposed PA provides a highly effective and competitive solution for next-generation ultra-wideband multifunction RF front-end applications.

## 2. GaN on SiC HEMTs

Owing to its high breakdown voltage, excellent thermal conductivity, and outstanding high-frequency properties, GaN results in far higher power densities and saturated output power than traditional semiconductor platforms such as GaAs or Si. As summarized in Table 1, GaN PAs consistently deliver superior efficiency, gain, and thermal performance, and they offer power-handling capabilities that surpass those achievable with GaAs- or even SiC-based devices.

**Figure 1:**
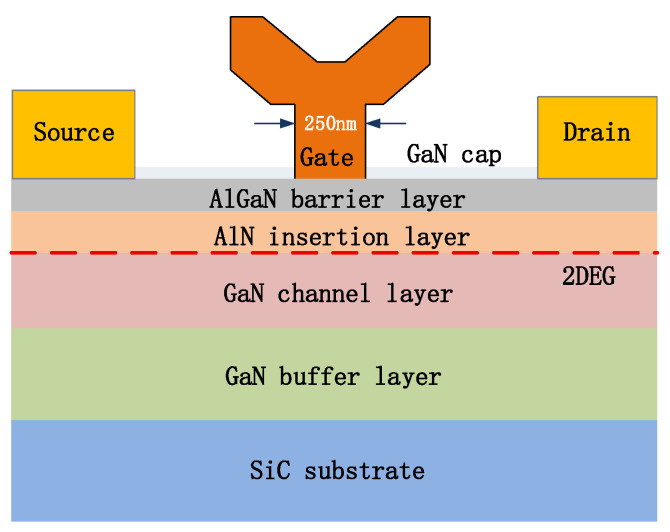
Schematic structure of the 250 nm GaN-SiC HEMT.

Figure 1 shows a simplified three-dimensional structure of an AlGaN/GaN HEMT. A high-density two-dimensional electron gas (2DEG) is produced at the AlGaN/GaN interface due to strong spontaneous and piezoelectric polarization. This polarization-induced charge enables a significantly higher sheet carrier density than in conventional III–V HEMTs, supporting superior power and high-frequency performance. To further enhance device performance, a thin GaN cap layer is commonly introduced. This cap layer improves gate control over the channel, leading to enhanced transconductance, increased saturation drain current, and reduced surface states and current collapse, which collectively help improve linearity and reliability.

**Figure 2:**
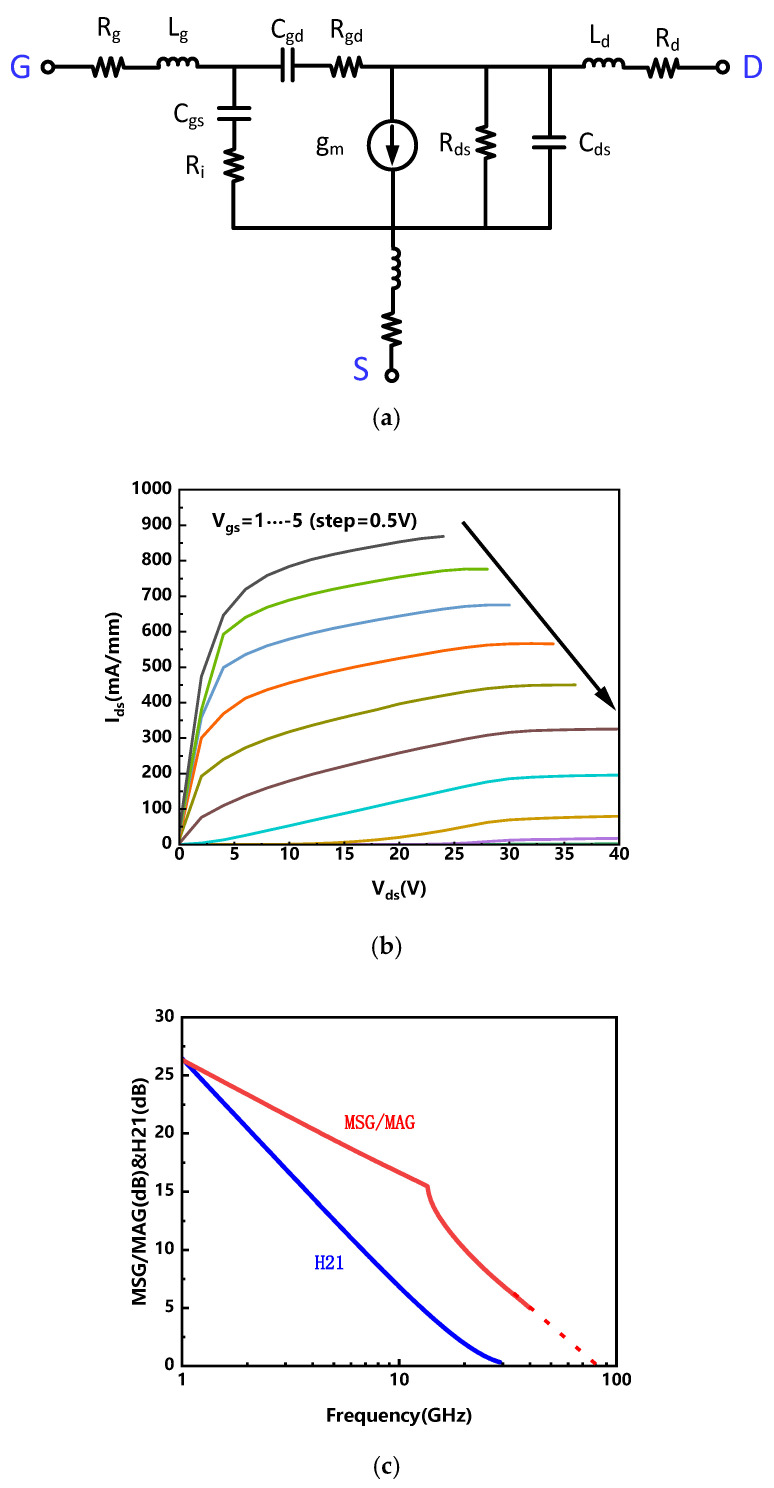
GaN HEMT characteristics: (**a**) equivalent small-signal circuit model; (**b**) measured output I–V characteristics; and (**c**) MSG/MAG and current gain ∣*H*_21_∣versus frequency.

The small-signal equivalent circuit of the GaN HEMT is shown in Figure 2a. In this model, Rs, Rd, and Rg represent the parasitic resistances of the source, drain, and gate, respectively, while C_gs_, C_gd_, and C_ds_ denote the parasitic capacitances between the corresponding terminals. L_g_, L_s_, and L_d_ represent the parasitic inductances associated with the gate, source, and drain leads. Analysis of the frequency response of a single transistor indicates that parasitic capacitances have little influence on gain at low frequencies. As the operating frequency increases, however, these parasitic effects become more pronounced, leading to gain degradation and bandwidth limitation. Therefore, improving the cut-off frequency typically requires the use of a transistor with a smaller effective size, which results in an inherent trade-off between bandwidth and gain. The output electrical behavior of the GaN HEMT is first examined through DC measurements, with Figure 2b presenting the measured I–V curve of an 8 × 100 μm device. To further evaluate RF performance, on-wafer, small-signal measurements were conducted, and the corresponding S-parameters were extracted. Based on these measurements, key gain-related figures of merit—maximum stable gain and maximum available gain—were derived, as summarized in Figure 2c. The extracted results indicate that the transistor can achieve a unity current gain frequency of 26 GHz and a maximum oscillation frequency of 90 GHz. Furthermore, the device demonstrates robust high-voltage capability, sustaining a drain–source breakdown voltage greater than 110 V while delivering a power density on the order of 4.5 W/mm.

## 3. Load-Pull and PA Design

The broadband power amplifier MMIC presented in this work was fabricated using a 0.25 μm GaN HEMT process. The typical device characteristics include a breakdown voltage of 120 V, a cutoff frequency of approximately 26 GHz, a saturation current density of about 700 mA/mm, and an output power density of around 4.5 W/mm at a drain bias of 28 V.

**Figure 3:**
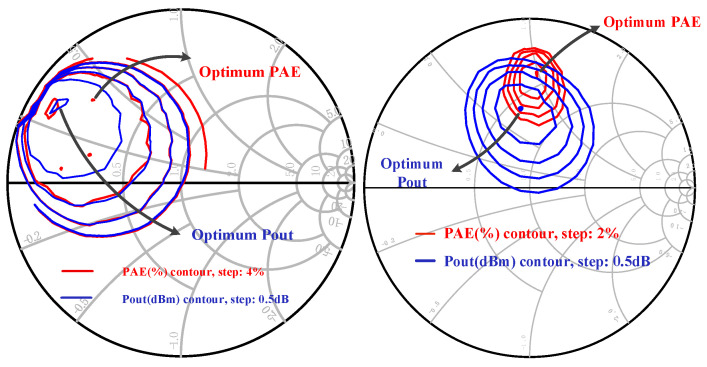
Source-pull and load-pull contours for output power (Pout) and power-added efficiency (PAE), together with the corresponding optimal impedances at the fundamental frequency.

To characterize the intrinsic capabilities of the aforementioned GaN HEMT, a load-pull simulation scenario was established in commercial computer-aided design (CAD) software. The transistor (8 × 100 μm) was biased in Class-AB (Vgs = −2 V, and Vds = 28 V). To accurately determine the optimal fundamental load conditions, large-signal load-pull simulations were performed at the target operating band. These simulations provide critical insights into the trade-off between output power and power-added efficiency and serve as the foundation for the subsequent design of the output-matching network. To achieve broadband high-efficiency matching, source-pull and load-pull simulations were first performed on the fundamental impedance of the final-stage 8 × 100 μm transistor. As illustrated in Figure 3, at 10 GHz, the optimal efficiency source impedance is 10 + j18 Ω, while the optimal efficiency load impedance is 19 + j49.5 Ω, corresponding to a maximum efficiency of approximately 56%.

**Figure 4:**
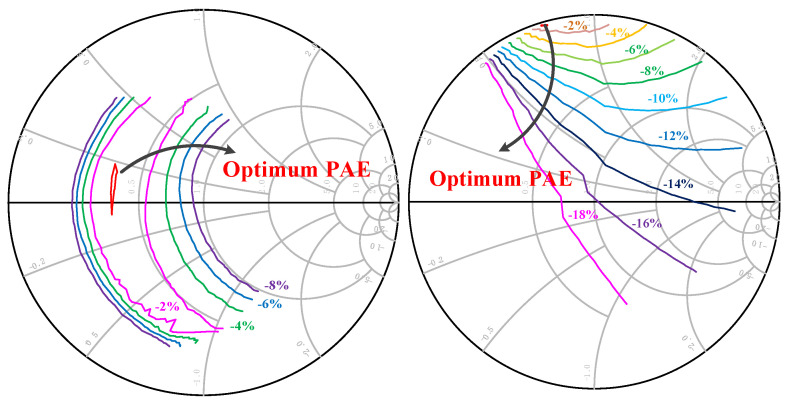
Source-pull and load-pull contours for power-added efficiency (PAE), along with the corresponding optimal impedances at the second-harmonic frequency.

Efficiency analysis of the power amplifier indicated that efficiency enhancement is not solely determined by fundamental impedance matching. Due to the presence of harmonic power components, harmonic impedance matching also plays a significant role, particularly under broadband conditions, where the second harmonic often falls within the operating frequency range. Therefore, considering harmonic impedance matching is of considerable importance. Figure 4 presents the source- and load-pull simulation results for the 8 × 100 μm transistor cell at the second-harmonic impedance points, for which fundamental impedance matching is assumed. The results show that when the second-harmonic impedance is optimized, the maximum efficiency increases from 57% to 73%, demonstrating that second-harmonic matching contributes substantially to efficiency improvement. Moreover, as observed in the load-pull contours in Figure 4, unlike the closed efficiency contours associated with the fundamental impedance, the second-harmonic contours exhibit a divergent distribution. This characteristic indicates a broader acceptable impedance region for the second harmonic; as long as the impedance lies within the high-impedance inductive region, high efficiency can be maintained. Such behavior greatly simplifies broadband second-harmonic matching.

**Figure 5:**
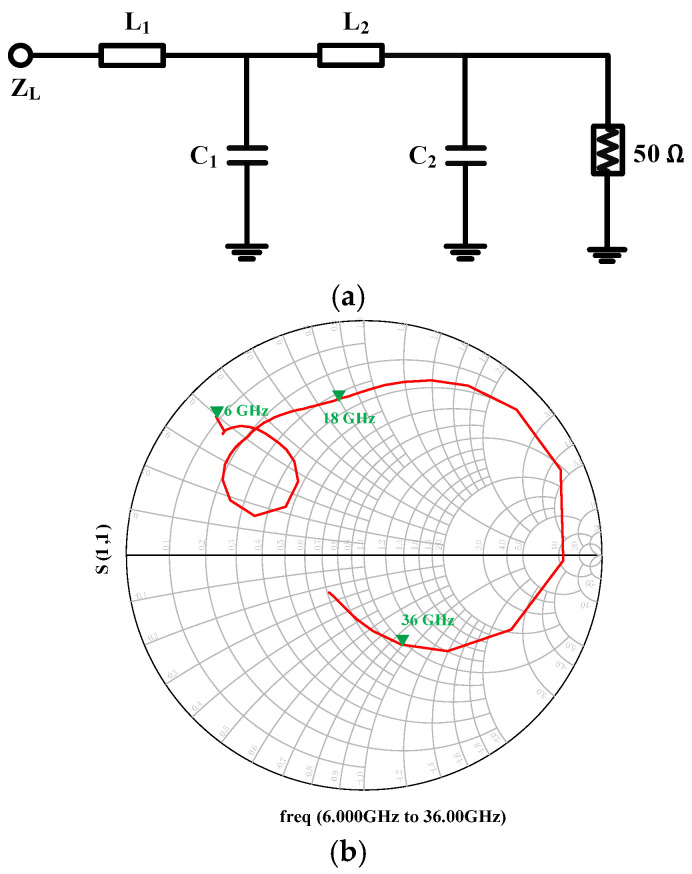
Chebyshev matching structure: (**a**) Circuit structure and (**b**) impedance trace.

To further enhance the large-signal efficiency of the proposed amplifier, harmonic impedance engineering at the output network is carefully incorporated. In particular, proper control of the second-harmonic impedance plays a critical role in shaping the voltage and current waveforms, thereby improving power-added efficiency (PAE) under broadband operation. Various matching strategies have been explored for second-harmonic impedance optimization. Among them, the Chebyshev low-pass filter is especially attractive due to its structural simplicity and low insertion loss, making it well suited for broadband output matching in power amplifier design. A schematic of a four-stage Chebyshev low-pass filter is shown in Figure 5a. Owing to the presence of shunt capacitors, the impedance locus at the second-harmonic frequency is shifted clockwise toward the high-efficiency region since the capacitive reactance 1/(jωC) decreases with an increasing frequency. This inherent frequency-dependent behavior enables simultaneous impedance control at both the fundamental and second-harmonic frequencies. To realize this behavior, a Chebyshev low-pass topology is employed for the output-matching network due to its capacity for broadband impedance transformation with controlled passband ripple. Considering an optimum fundamental load impedance of $Z_{L}=19+j49.5\;\Omega$ at 10 GHz, the real part (19 Ω) is transformed to a standard 50 Ω load, corresponding to a real-to-real impedance transformation ratio of r=2.5. Based on the synthesis method described in ref. [18], a fourth-order Chebyshev low-pass prototype, as shown in Figure 5a, is constructed. The normalized prototype parameters are then determined according to this transformation ratio using standard design tables. The element values are expressed as follows:

$$L_{n}=\frac{1}{g_{2n-1}}\cdot \frac{\omega_{0}^{\prime }}{\omega_{0}}\cdot \frac{50}{r},\;C_{n}=g_{2n}\cdot \frac{\omega_{0}^{\prime }}{\omega_{0}}\cdot \frac{r}{50}$$

where $\omega_{0}^{\prime }=1$, $\omega_{0}$ denotes the angular frequency at 12 GHz, and r represents the impedance transformation ratio. Subsequently, a real-to-complex Chebyshev low-pass matching network is further optimized to accurately match the complex-valued $Z_{L}$. The lumped element values obtained from above equations are employed as initial conditions, and a post-optimization procedure is carried out to achieve the real-to-complex transformation, as described in ref. [19].

Figure 5b illustrates the impedance trajectories of the proposed output-matching network based on the Chebyshev topology. It can be observed that, over the 6–18 GHz fundamental frequency band, the impedance points remain close to the optimum efficiency region. Meanwhile, across the 18–36 GHz second-harmonic band, the impedance trajectories are distributed around the high-efficiency region. These results demonstrate that the proposed broadband matching strategy achieves concurrent impedance optimization at both the fundamental and second-harmonic frequencies, thereby providing an effective solution for high-efficiency wideband power amplifier design and establishing a solid basis for the subsequent experimental validation.

**Figure 6:**
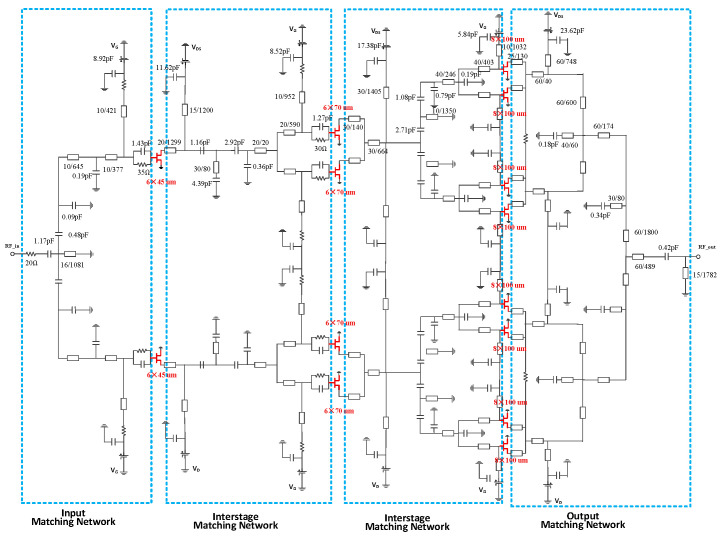
Schematic of the proposed 6–18 GHz MMIC power amplifier employing a three-stage architecture with broadband resistive–reactive matching networks.

Figure 6 is a schematic of the proposed MMIC PA. The design employs a three-stage common-source architecture, incorporating a hybrid resistive–reactive matching network to facilitate wideband performance. The first stage is designed as a driver stage to provide adequate small-signal gain and favorable input matching. The second stage serves as an interstage amplifier, ensuring sufficient driving capability while maintaining gain flatness across the targeted frequency band. The final stage functions as the power stage and employs eight parallel transistor cells to enhance the output power capability.

Broadband matching networks are incorporated at the input, between stages, and at the output of the amplifier. These matching networks are implemented using a combination of transmission-line sections and lumped passive components to compensate for device parasitic effects and facilitate wideband impedance transformation. The interstage matching networks are optimized to ensure smooth impedance transition between adjacent stages and improve the overall stability of the amplifier. The output-matching network is optimized under large-signal operating conditions in order to achieve a favorable trade-off between output power and power-added efficiency (PAE) over the entire operating bandwidth. It is worth emphasizing that the parallel transistor configuration in the final stage increases the effective gate periphery and current handling capability. Moreover, the symmetric circuit topology and balanced power-combining structure contribute to uniform current distribution and improved thermal behavior, thereby enhancing the stability and reliability of the proposed amplifier. The series and parallel inductors used in the circuit design are implemented using microstrip lines. Interstage and output-matching circuits into which high-drain DC current flows were designed using double-layered metal lines to withstand high current and minimize line loss. TaN resistors of 30 Ω are used between transistor cells to prevent plausible odd-mode oscillations that may be caused by transistor imbalance in the corporate structure [20,21]. Following the completion of the circuit schematic, a fabrication-oriented MMIC layout was developed to accurately reflect the schematic design, and full electromagnetic (EM) verification was subsequently performed to validate the layout performance. The transition from schematic to layout was carried out iteratively under the guidance of EM simulations. In practice, the physical layout inevitably introduces nonideal effects that are not captured at the schematic level, including microstrip parasitics, interconnect coupling, and unintended radiation, which may cause the electromagnetic behavior of the chip to deviate from ideal circuit predictions. Therefore, the layout is continuously tuned and optimized based on EM simulation results to achieve close agreement between layout-level and schematic-level performance, suppress parasitic electromagnetic effects, and ultimately ensure that the fabricated chip meets the specified design requirements.

## 4. Fabrication and Measurement Results

**Figure 7:**
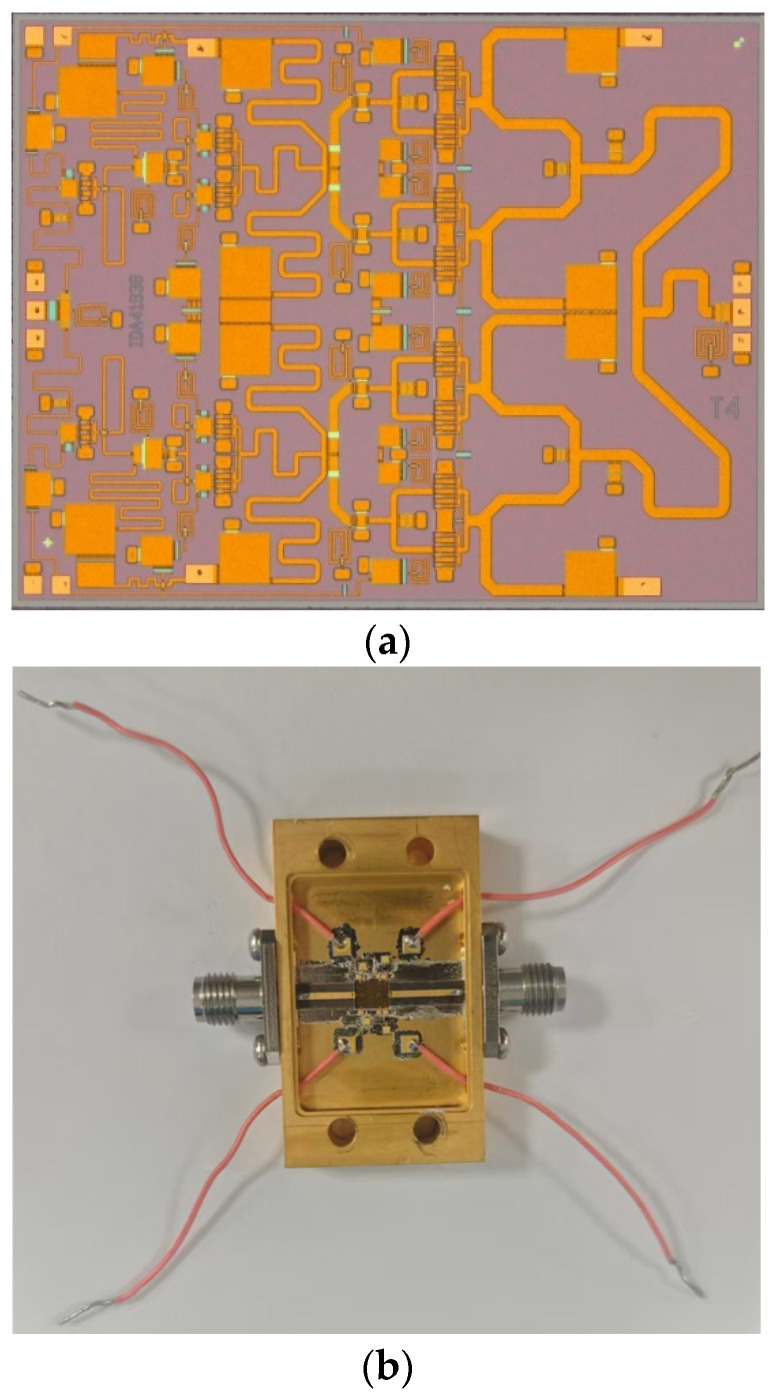
Photograph of the fabricated chip for the 6–18 GHz GaN power amplifier we designed. (**a**) Chip. (**b**) Test jig.

**Figure 8:**
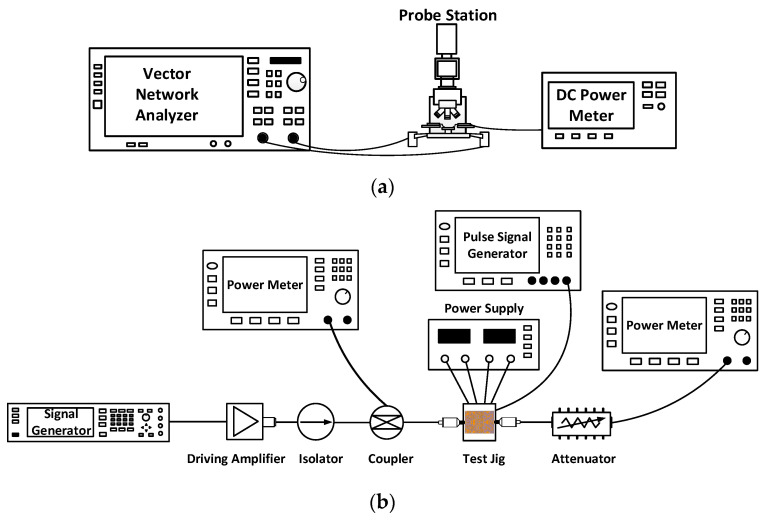
Measurement setup block diagrams: (**a**) small-signal and (**b**) large-signal.

To verify the validity of the proposed design, the MMIC was fabricated using a 4-inch 0.25 μm GaN HEMT process. The fabricated chip occupies an area of 4 × 3.2 mm^2^, with a substrate thickness of 80 μm, and a photomicrograph of the chip is shown in Figure 7. After fabrication, on-wafer measurements were performed using a vector network analyzer (VNA) and a power meter in conjunction with high-power ground–signal–ground (G–S–G) probes. The small-signal measurement configurations are illustrated in Figure 8a. S-parameter measurements were carried out on-wafer using a probe station connected to a VNA. To suppress self-heating and the associated performance degradation, pulsed biasing was applied to the device under testing (DUT) via an external pulse generator during measurement.

For power characterization, the MMIC chip was eutectically bonded onto a 0.2 mm thick diamond–copper composite carrier to enhance heat dissipation, and it was then soldered onto a copper test jig. The RF input and output ports of the MMIC were connected to 0.254 mm thick Duroid 5880 microstrip lines using gold wire bonding, while the RF signals were routed through coaxial connectors mounted on both sides of the test fixture. To ensure stable biasing and suppress RF interference, 100 and 1000 pF decoupling chip capacitors were connected in parallel at both the gate and drain bias nodes, effectively filtering supply noise and preventing RF leakage into the DC bias paths. This packaging scheme ensures efficient heat dissipation and robust RF grounding without additional thermal management structures. Figure 8b illustrates the large-signal measurement setup. The measurements were conducted under pulsed operation at an ambient temperature of 25 °C. The input signal was generated by a signal generator and further amplified by a driver amplifier to provide sufficient excitation power. An isolator was inserted between the driver amplifier and the power amplifier (PA) to suppress reflections and enhance measurement stability. For system calibration, the reference planes were established at the input and output ports of the power amplifier (PA), enabling de-embedding of external components—including cables, amplifiers, and directional couplers—and thereby ensuring accurate extraction of intrinsic device performance. At the output stage, a fixed attenuator was placed between the PA and the spectrum analyzer to keep the measured signal within the instrument’s dynamic range and prevent potential damage due to excessive power levels. Additional isolation was incorporated where necessary to further suppress reflections and improve overall measurement accuracy and reliability.

**Figure 9:**
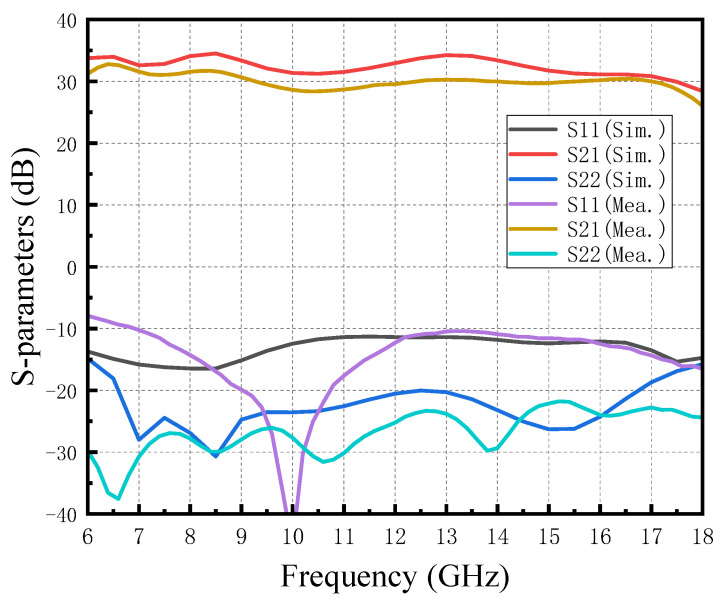
Small S-parameters of 6–18 GHz power amplifier.

Figure 9 presents the measured small-signal S-parameters, with the power amplifier biased at $V_{G}=-2\;V$ and $V_{D}=28\;V$. Good agreement can be observed between the simulated and measured results. Figure 8 shows a comparison of the simulated and measured S-parameters over the 6–18 GHz frequency range. The measured small-signal gain (S_21_) ranges from 26 dB to 32 dB across this band.

**Figure 10:**
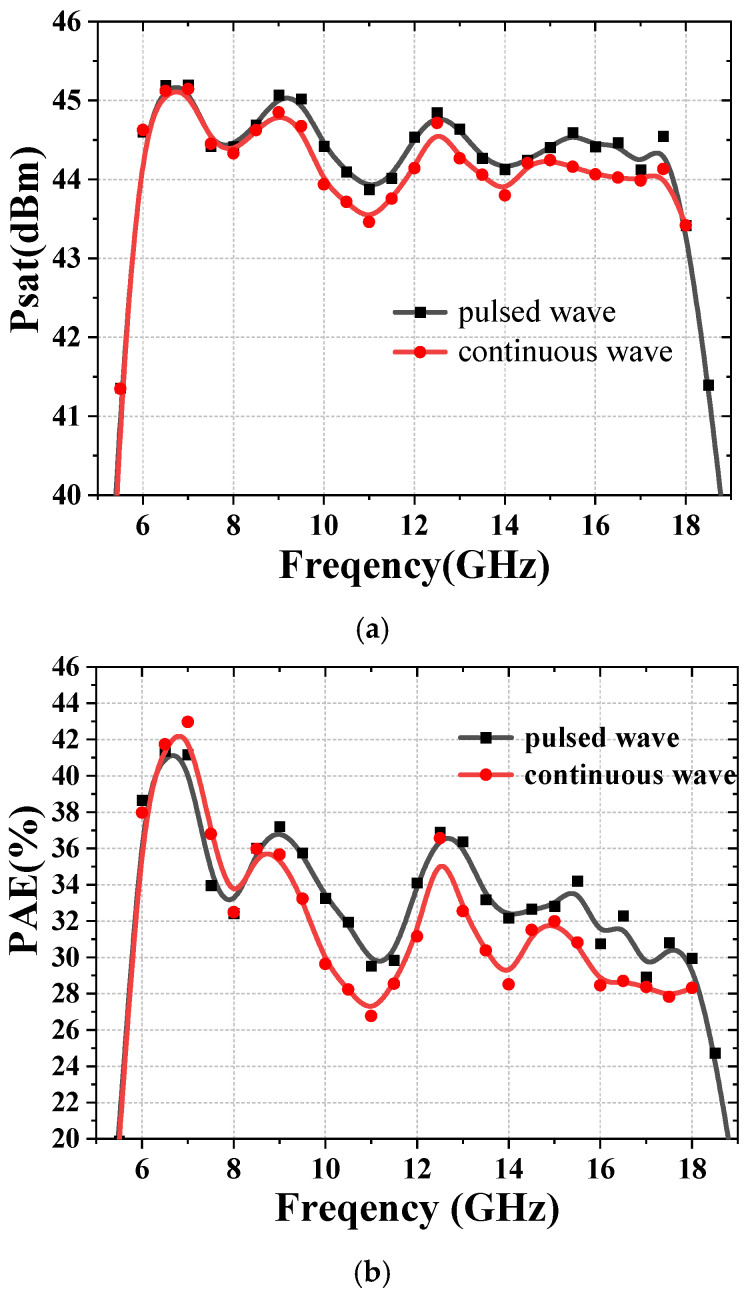
Measured large-signal performance of the proposed 6–18 GHz power amplifier under pulsed and continuous-wave (CW) operation: (**a**) output power and (**b**) power-added efficiency (PAE) versus frequency.

Figure 10 illustrates the large-signal performance under continuous-wave and pulsed conditions (VDS = 28 V, 100-μs pulse width, 10% duty cycle), showing saturated output power and high power-added efficiency (PAE) over the operating frequency range. Under pulsed wave (CW) operation, an output power of 44 to 45 dBm and a PAE of 30 to 40% were found, while the output power was measured to be 43.8 to 45 with an associated PAE of 28% to 40% in 6–18 GHz under continuous-wave conditions. Compared to pulsed operation, the output power reduced by approximately 0.3 dB, and the average PAE decreased by about 2%, with more pronounced degradation at higher frequencies. This degradation is primarily attributed to significant heat accumulation under CW excitation, which raises chip temperature, increases the channel resistance of the HEMT devices, and consequently reduces the saturation output power.

**Figure 11:**
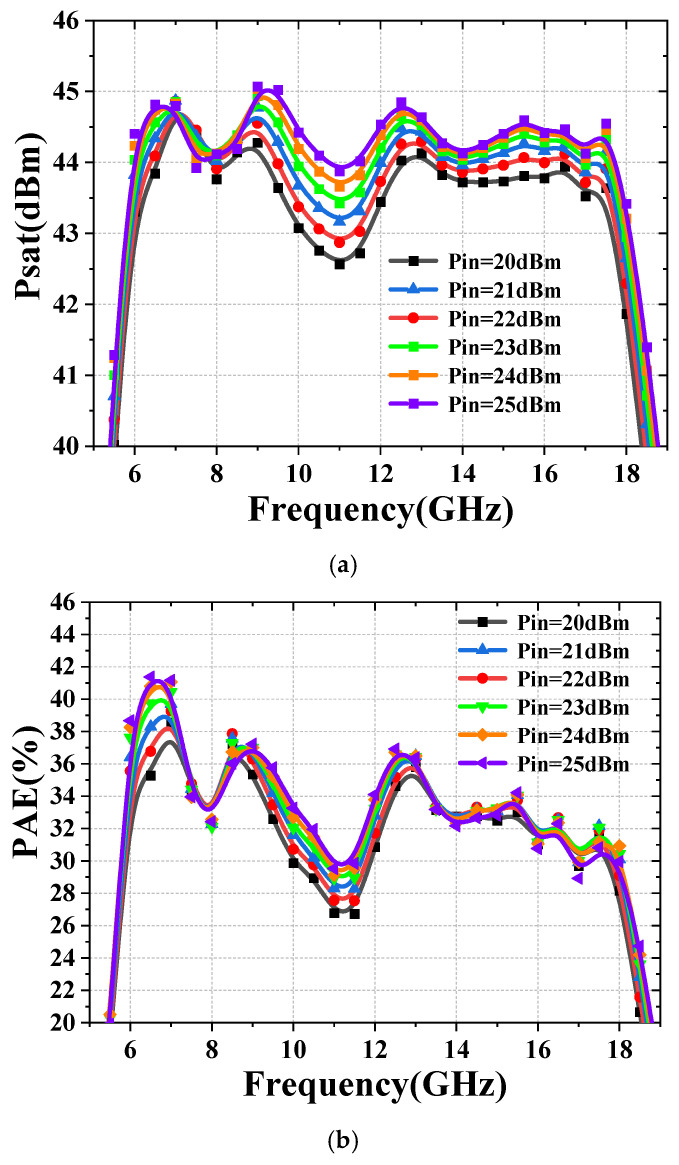
Measured large-signal performance of the 6–18 GHz power amplifier: (**a**) output power and (**b**) power-added efficiency (PAE) as functions of input power.

Figure 11 shows the large-signal output characteristics of the power amplifier under varying input power levels. The DC bias conditions were identical to those used in the small-signal test. A pulsed drain modulation method was employed, with a pulse width of 100 μs and a duty cycle of 10%. When the input power reaches 25 dBm, the amplifier delivers an output power exceeding 43.5 dBm across the entire operating band while maintaining an average efficiency of approximately 30%, which highlights its ability to sustain both high output power and efficiency under broadband operating conditions.

**Figure 12:**
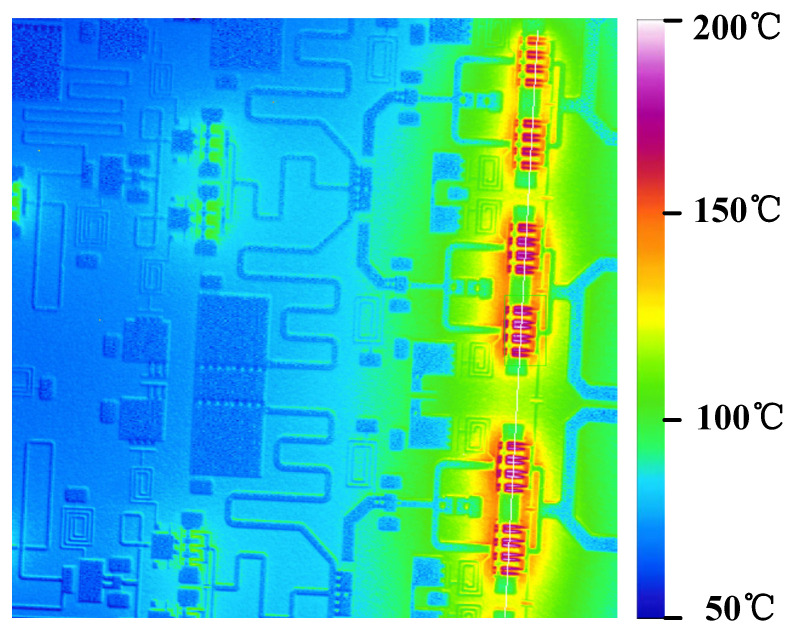
Chip temperature distribution of the 6–18 GHz power amplifier MMIC.

Infrared thermographic measurements were performed on the MMIC under both continuous-wave (CW) and pulsed-bias operating conditions. Figure 12 illustrates the thermal behavior of the chip for pulsed and CW excitations. The measurements were carried out at 11 GHz with an input power of 25 dBm. During CW operation, the overall chip temperature increased significantly, and the peak temperature rose to nearly 190 °C. This elevated thermal stress adversely affects transistor characteristics, resulting in a reduction in output power and a noticeable degradation in power-added efficiency (PAE). The thermal resistance (*θ*) is defined as the ratio of the temperature rise (ΔT) to the dissipated power, given by

$$\theta =\frac{∆T}{P_{diss}}$$

where $P_{\mathrm{diss}}=P_{DC}-P_{out}$.

Here, ΔT is the temperature difference between the peak channel temperature of the GaN device ($T_{c}$) and the temperature of the test fixture ($T_{j}$). Under continuous-wave (CW) operation, the extracted thermal resistance is 2.3 °C/W. It is worth emphasizing that this value lies well within the safe operating limits of the employed GaN technology, indicating effective heat dissipation. This result further confirms the thermal reliability and robustness of the proposed design under high-power operating conditions.

Table 2 provides a comprehensive comparison between the proposed design and representative 6–18 GHz power amplifier (PA) MMICs reported in the recent literature. To evaluate the overall performance, a unified Figure of Merit (*FoM*) is adopted [22], which is defined as follows:

$$FoM=P_{out}(w)\cdot G_{P}\cdot PAE\cdot f^{2}$$

where *P_out_*, *Gp*, *PAE*, and *f*_0_ denote the saturated output power, power gain, power-added efficiency, and operating frequency, respectively. It is worth noting that, for the *FoM* calculation, the *PAE* and *P_out_* employed in the formulation are taken to be the minimum values over the entire 6–18 GHz frequency band, thereby ensuring conservative and rigorous performance evaluation. By incorporating an output-matching network with impedance-tuning stubs engineered at both the fundamental and second-harmonic frequencies, the proposed PA attains enhanced output power while preserving a compact chip footprint. The proposed amplifier can achieve a saturated output power higher than most recently reported designs, with only Ref. [23] showing a marginal advantage (~0.2 dB). In terms of *PAE*, it outperforms nearly all prior approaches and maintains a higher average efficiency across the operating band, demonstrating superior broadband efficiency uniformity. More importantly, the proposed design simultaneously achieves high output power and high efficiency, effectively alleviating the conventional trade-off observed in prior work. Furthermore, the introduced figure of merit (*FoM*) indicates that our design achieved the highest value among the reported designs, quantitatively confirming its advantage. These results validate the effectiveness of the proposed co-design methodology integrating broadband matching and harmonic impedance engineering.

## 5. Conclusions

In this work, a wideband, high-efficiency GaN MMIC power amplifier is presented. By incorporating a low-loss low-pass matching network that simultaneously optimizes the impedances at both the fundamental and harmonic frequencies, the proposed design achieves enhanced broadband efficiency. A 6–18 GHz push–pull power amplifier was implemented using a 0.25 μm GaN HEMT technology, delivering a saturated output power of 43.5 dBm while maintaining a power-added efficiency exceeding 30% across the entire operating band. Compared with previously reported 6–18 GHz GaN power amplifiers, the proposed PA demonstrates superior performance, validating the effectiveness of the proposed broadband impedance-matching strategy for high-efficiency wideband applications. Owing to its wide bandwidth and high output power capability, the proposed MMIC is well suited for integration into broadband high-power transmission–reception front-end modules.

## Figures and Tables

**Table 1:** Electronic and physical properties of GaN compared with Si, SiC and GaAs [16,17].

Property	GaAs	Si	SiC	GaN
Bandgap (eV)	1.4	1.12	3.2	3.4
Breakdown field Ebr [mV/cm]	0.4	0.3	3.5	3.3
Electron mobility μn [cm^2^/V·s]	8500	1500	650	2000
Hole mobility [cm^2^/V·s]	400	480	120	300
Transit frequency *f*_t_ (GHz)	150	20	20	150
Saturation electron drift velocity *v*_sat_ (cm/s)	1.2 × 10^7^	107	2.7 × 10^7^	2.5 × 10^7^
Dielectric constant	10	11.9	12.5	9.5
Thermal conductivity K [W/(m·K)]	450	150	550	130

**Table 2:** Performance comparison of 6–18 broadband PAs.

Year, Ref.	Process	Operation	Saturated Output Power *P_out_* (dBm)	*PAE*/%	Gain (dB)	*G_P_* (dB)	Size (mm^2^)	*FoM*
2010, [8]	GaN	CW	40	20	20	17	6.43 × 3.08	14,434
2013, [9]	GaN	CW/pulse 10%	40.2/40.9	18/19	18	12.2/15	5.5 × 3.5	4504
2025, [10]	GaN	CW	41.1	18.8	19.7	/	3.7 × 3.9	/
2016, [11]	GaN	CW/pulse 10%	43/44	14	13	11	7.8 × 2.7	5075
2018, [12]	GaN	CW	40.2/40.6	15.5/15	15.3/16.7	11	2.1 × 5.1/2.1 × 5.1	2942
2023, [13]	GaN	CW	41	19	24	15	4 × 3	10,893
2015, [14]	GaN	CW	35	13	16	/	3.385 × 2.59	/
2025, [15]	GaN	CW	12.5	19	25	17.2	3.7 × 4.2	26
2026, [24]	GaN	CW	40.8	27–38	25	18	4.5 × 3.4	29,439
2026, [25]	GaN	CW	40.2	24–36	25	19.7	4.1 × 3.7	33,801
2026, [23]	GaN	CW	43.7	23.6–40.4	25	16.8	3.2 × 2.9	38,130
This work	GaN	CW	43.5	27–41	30	17	4 × 3.2	43,629

## Data Availability

The original contributions presented in this study are included in the article. Further inquiries can be directed to the corresponding author.
